# Designing a system for performance appraisal: balancing physicians’ accountability and professional development

**DOI:** 10.1186/s12913-021-06818-1

**Published:** 2021-08-12

**Authors:** Elisa Bindels, Benjamin Boerebach, Renée Scheepers, Annemiek Nooteboom, Albert Scherpbier, Sylvia Heeneman, Kiki Lombarts

**Affiliations:** 1 Department of Medical Psychology, Amsterdam Center for Professional Performance and Compassionate Care, Amsterdam University Medical Centers, Amsterdam, the Netherlands; 2 Department of Educational Development and Research, Faculty of Health, Medicine and Life Sciences, Maastricht University, Maastricht, the Netherlands; 3 Department of Clinical Psychology, Faculty of Social and Behavioral Sciences, Utrecht University, Heidelberglaan 1, H1, 54, 3584 CS Utrecht, the Netherlands; 4 Department of Value-Based Healthcare, St. Antonius Hospital, Nieuwegein, the Netherlands; 5 Department of Socio-Medical Sciences, Erasmus School of Health Policy and Management, Erasmus University of Rotterdam, Rotterdam, the Netherlands; 6 Department of Intensive Care, Amsterdam University Medical Centers, Amsterdam, the Netherlands; 7 Nooteboom Consult, Amsterdam, the Netherlands; 8 Department of Educational Development and Research, Faculty of Health, Medicine and Life Sciences, Maastricht University, Maastricht, the Netherlands; 9 Department of Pathology, Faculty of Health, Medicine and Life Sciences, Maastricht University, Maastricht, the Netherlands

**Keywords:** Continuing professional development (CPD), Revalidation, Maintenance of certification (MoC), Re-registration, Performance appraisal and assessment, Multisource feedback (MSF), Coaching, Design-based research (DBR)

## Abstract

**Background:**

In many healthcare systems, physicians are accustomed to periodically participate in individual performance appraisals to guide their professional development. For the purpose of revalidation, or maintenance of certification, they need to demonstrate that they have engaged with the outcomes of these appraisals. The combination of taking ownership in professional development and meeting accountability requirements may cause undesirable interference of purposes. To support physicians in their professional development, new Dutch legislation requires that they discuss their performance data with a non-hierarchical (peer)coach and draft a personal development plan. In this study, we report on the design of this system for performance appraisal in a Dutch academic medical center.

**Methods:**

Using a design-based research approach, a hospital-based research group had the lead in drafting and implementing a performance appraisal protocol, selecting a multisource feedback tool, co-developing and piloting a coaching approach, implementing a planning tool, recruiting peer coaches and facilitating their training and peer group debriefings.

**Results:**

The system consisted of a two-hour peer-to-peer conversation based on the principles of appreciative inquiry and solution-focused coaching. Sessions were rated as highly motivating, development-oriented, concrete and valuable. Peer coaches were considered suitable, although occasionally physicians preferred a professional coach because of their expertise. The system honored both accountability and professional development purposes. By integrating the performance appraisal system with an already existing internal performance system, physicians were enabled to openly and safely discuss their professional development with a peer, while also being supported by their superior in their self-defined developmental goals. Although the peer-to-peer conversation was mandatory and participation in the process was documented, it was up to the physician whether or not to share its results with others, including their superior.

**Conclusions:**

In the context of mandatory revalidation, professional development can be supported when the appraisal process involves three characteristics: the appraisal process is appreciative and explores developmental opportunities; coaches are trustworthy and skilled; and the physician has control over the disclosure of the appraisal output. Although the peer-to-peer conversations were positively evaluated, the effects on physicians’ professional development have yet to be investigated in longitudinal research designs.

**Supplementary Information:**

The online version contains supplementary material available at 10.1186/s12913-021-06818-1.

## Background

For the purpose of both continuing professional development (CPD) and accountability to the public, in many western countries physicians periodically participate in performance appraisals. Performance appraisal is an educational intervention, which involves a formative conversation between two professionals. Originally, the appraisal process was intended to provide a physician with feedback on his or her performance, map a physician’s progress and identify areas for further development. Performance appraisal was not designed as an assessment of competence which a physician would either pass or fail [1]. In response to society’s call for transparency however, performance appraisal has been incorporated into procedures for revalidation (UK), recertification (USA, Canada) and re-registration (Netherlands). In this context, participation in performance appraisal is assumed to provide more objective assurance that a physician is up to date and fit to practice medicine [2–4]. The interlinking of appraisal and revalidation presents itself as a delicate matter, as this may cause undesirable interference of two types of goals: professional development versus accountability. Physicians may perceive these goals as conflicting, framing the appraisal process as summative (i.e. to detect and weed out bad apples) instead of formative (i.e. to raise standards and support professional development) [5, 6]. The literature indicates that this interference may jeopardize physicians’ engagement in the appraisal process, since mandatory accountability and transparency requirements seem principally incompatible with the needs for psychological safety and intrinsic motivation. Care must be taken that the appraisal process is experienced and used as an opportunity for learning and development and is not turned into a tick-box-exercise, associated with a loss of personal investment and a disposition of compliance.

Up till now, no consensus exists on the appropriate incorporation of performance appraisal into revalidation procedures. Some countries have no formal process in place, while others rely heavily on the collection of credits for continuing learning and development activities [7]. Nevertheless, there is a growing interest in the use of multisource feedback (MSF) for performance appraisal purposes, and regulatory bodies in the USA, Canada, the UK and the Netherlands use MSF as part of revalidation and evaluation programs for practicing physicians [8–10]. Feedback from peers, co-workers and residents is found to be necessary to inform physicians’ self-assessment by providing a more realistic view of how they perform [11]. Research on how physicians use MSF, however, has demonstrated that this feedback does not self-evidently find its way into performance change. Feedback can be perceived as disconfirming or disappointing, it may evoke an emotional reaction that can interfere with the ability to assimilate and learn from it, or the feedback may not be specific enough to catalyze certain performance changes [10]. Also, one’s self-efficacy and motivation and the collegial culture can influence the process of using feedback for learning and change. Reflective discussions guided by another person within a respected, engaged relationship (a peer, coach or mentor) can foster feedback reconciliation with one’s own self-assessment and promote growth through facilitating feedback acceptance and use and offering appropriate challenge. Coaching strategies can support personal and professional development by guiding the feedback recipient in identifying their own needs and goals and developing a realistic action plan [12, 13]. The addition of ‘coaching’ to the feedback lexicon is significant, because it places feedback in a different light. Coaching is developmental, and philosophically it moves feedback away from its historic tie to assessment and moves it towards learning [13]. Feedback, in combination with coaching, can be a powerful strategy for fostering physicians’ continued development and growth. More broadly, it can serve to support physicians in their complex professional roles and their capacity to be self-directed yet function capably as team member, to make decisions on their own yet know when to ask for help, and to maintain their own health and sense of well-being in the presence of multiple system and other demands [14]. Reports of alarming levels of stress and burnout among physicians direct our attention to ways of supporting physicians for these complicated roles and nurture their resilience [15].

In the Netherlands, the requirements for re-registration have recently changed; new legislation concerning physicians’ individual performance appraisal has been adopted. As per 2020, practicing physicians are required to five-yearly collect performance feedback, discuss their feedback with a trained, non-hierarchical facilitator and define and follow-up on a personal development plan (PDP) [16]. The aforementioned concern over the two types of potentially conflicting goals of re-registration and performance appraisal, however, may jeopardize this appraisal process. As mentioned earlier, mandatory accountability and transparency requirements seem principally incompatible with the needs for psychological safety and intrinsic motivation. In this study, we report on the design of an individual performance appraisal system in a Dutch academic medical center, in which this incompatibility was taken into account. The design problem was formulated as follows: how to design a system that will successfully comply with both revalidation requirements and the need for professional development? By using a design-based research (DBR) approach, our aim was to define, build and implement a system around characteristics that are crucial for the facilitation of professional development in the context of mandatory re-registration. Since MSF programs do not traditionally involve coaching elements, the design presented in this study is among the first attempts to combine MSF and coaching. We aim for this design to serve as an inspiring example for other institutions or health care systems dealing with the same challenge. Before we will discuss the method however, we will first provide relevant background information on the re-registration procedure and appraisal systemin the Netherlands.

## Policy background

### Requirements for re-registration in the Netherlands

After completion of residency training, physicians in the Netherlands are registered with the Registration Committee of Medical Specialists. In order to maintain their license, physicians need to meet a number of re-registration requirements, which is assessed once every five years. These involve (1) working in clinical practice for a minimum of 16 h per week, (2) participating in accredited expertise-promoting activities equaling a minimum of 40 hours per year, and (3) participating in the external peer review (called *visitatie*) program of a physician’s professional body. The latter national program focuses on physicians’ group performance, assessed in highly protocolled peer site-visits. Participation in the *visitatie* program is mandatory since 1995. Since the performance of *individual *physicians is not the primary focus of the *visitatie *program, the Dutch medical regulatory and registration authorities recently added a fourth re-registration requirement. As per 2020, physicians (4) must demonstrably work on their individual professional development [16]. This new requirement is referred to as the IFMS (Individual Functioning of Medical Specialist) requirement [4].

### Performance appraisal: the IFMS system

The IFMS system is based on research and is built on previous designs of appraisal systems used in the Netherlands. In the period 2005–2007, the first IFMS system was developed in a collaborative project with the Dutch National Organization of Medical Specialists (Orde van Medisch Specialisten or OMS), the Dutch Institute of Quality Improvement (CBO), 8 medical professional societies and 8 pilot (non-academic) hospitals. One of the authors (KL) was the national advisor and co-project leader. The development, implementation and evaluation have been accounted for in a PhD thesis [17] and policy recommendations for the future of IFMS were published by the national IFMS committee of the OMS [18]. Three instruments, which were developed in Canada, the US and the UK, were examined for suitability in the Dutch context [19]. The first two instruments, the Canadian method of multisource feedback (MSF) [20] and the American method of Peer Associate Rating (PAR) [21] both used structured questionnaires to collect information about a physician’s individual performance. These questionnaires were presented to persons with whom the physician under evaluation has a close working relationship, that is physician-colleagues, staff, residents and patients for the Canadian method and physician-colleagues for the American method. The third instrument, the British method of Appraisal & Assessment (A&A) [22], concerns a qualitative performance assessment posing 3 open-ended questions to a limited number of the physician’s peers and coworkers. For the final Dutch IFMS system, the MSF methodology and the A&A methodology were combined by using quantitative questionnaires with room for qualitative feedback [23]. The IFMS requirement consists of a five-yearly evaluation of individual performance and dictates the use of multisource feedback (MSF). The procedure entails three consecutive steps: (1) take part in the collection of MSF, (2) discuss the feedback with a trained facilitator with whom there is no hierarchical or otherwise dependent relationship, and (3) define and yearly evaluate/adjust a personal development plan (PDP). Every physician must maintain a portfolio in which he or she shows evidence of his or her professional performance activities and outcomes covering all professional (medical and generic) competencies [24]. The objective of the IFMS requirement is to maintain or enhance the quality of physicians´ individual performance; its purpose is not to identify malfunctioning physicians. Other systems are available to manage (suspected) malfunctioning or poor performance [4].

### Performance appraisal: the IFMS system in academic medical centers

In the Netherlands, physicians carry out their professional duties in various organizational and legal contexts. Physicians can be independent entrepreneurs organized in ‘medical specialist companies’ through which specialist care is contracted by hospitals, or physicians are employed by hospitals. At this point, an important difference between physicians working in academic and non-academic settings must be noted. Physicians working in academic medical centers are employed by the hospital, and therefore they are obliged to demonstrate accountability for their performance to the hospital. Within the hierarchical structure of academic medical centers, there has long been an existing procedure involving an annual review of the physician’s individual performance. The annual review is executed by the physician´s hierarchical superior, i.e. head of department (HoD), and provides a means to review the physician’s individual career development in alignment with the strategic objectives of the hospital. This annual review allows for customization of individual development, but does not take into account actual performance feedback from peers and coworkers [7]. The initial IFMS system, as described in the previous section, was not developed for use in academic medical centers and was not mandatory for individual re-registration purposes. The new context in which participation in IFMS is mandated for re-registration posed a brand new situation, requiring a new design. The new to be designed and implemented IFMS system must be embedded within the existing structure of annual reviews. Redundancy between the IFMS procedure and the procedure of annual reviews had to be prevented or at least minimized. On the one hand, the physician needs to be able to safely discuss his or her performance with a trained, non-hierarchical facilitator; on the other hand, it must be assured that the physician can be supported by his or her superior in intended developmental activities. The present study is about the design of the IFMS system in one specific hospital, the Academic Medical Center (AMC). After the development and implementation of this IFMS system was completed, the AMC was merged with VU University Medical Center and continued as one organization under the new name Amsterdam University Medical Centers (Amsterdam UMC).

## Method

### Study design

To design, build, and implement the IFMS system in the Academic Medical Center that would honor both accountability and professional development purposes, we adopted a design-based research (DBR) approach. DBR is a fruitful approach for the (re)design of work-based learning environments and assessment programs. The five important characteristics of DBR are the following: (1) it takes place in continuous cycles of design, evaluation and redesign; (2) it takes place in an authentic practice context; (3) it is aimed both at testing and refining theories and also advancing practice; (4) it is a methodologically diverse and operation-oriented process; (5) designers, researchers and practitioners with different expertise interact frequently and share their ideas [25]. Within design-based research, four phases can be distinguished: (1) analysis and exploration, (2) design and construction, (3) evaluation and reflection, and (4) implementation and spread [26]. In the case of the IFMS system in the Academic Medical Center, the research team first began to clarify the design problem and set up a fruitful collaboration with the main stakeholders. Second, the research team worked together with the stakeholders to articulate the design principles or criteria to be taken into account. Third, the research team empirically investigated design ideas on a small scale and reflected on the findings to adapt and strengthen the overall design. Fourth, the research team involved various stakeholders to tailor the new system to the specific hospital context and scale up the implementation process. In the following two sections, we will elaborate on the role and composition of the research team, the stakeholders involved and the different steps in the design, evaluation and redesign of the IFMS system.

### Research team and stakeholders

The Executive Board of the hospital assigned the design of the IFMS system, from protocol development through evaluation and implementation, to a hospital-based research group. This research group, named Professional Performance and Compassionate Care (PP&CC), has extensive experience in physicians’ performance evaluation and improvement (www.professionalperformance-amsterdam.com). Four members of PP&CC (KL, RS, BB and EB) formed the project team, all also co-authoring this paper. The other two authors (SH, AS) have extensive experience in educational research and development and acted as a sounding board in the conception and drafting of this article. In 2016 the project team sought the opinions of both internal and external experts on medical leadership, hospital administration and representatives of the Dutch medical regulatory and registration authorities. This was done to assure a good position of the IFMS system within the organizational context of the hospital. Expertise from a psychologist (AN) was sought to develop an approach to discuss the performance feedback, which would facilitate physicians’ professional development. The hospital’s physician group of (17) neurologists was found willing to pilot this approach. Involvement of various stakeholders was essential to tailor the new system to the specific hospital context. For example, coordination with department heads was needed to let the IFMS system fit in with the existing procedure of annual reviews.

### Design, evaluation and redesign

A cyclic process of defining underpinning design principles, evaluation activities and refinement was the basis for the development of the IFMS procedure in the AMC. The process consisted of five steps:

1. Development of an IFMS protocol;
2. Selection of an MSF tool;
3. Development, piloting and evaluation of a coaching approach;
4. Recruitment, training and evaluation of coaches;
5. Hospital-wide implementation..

At the beginning of the design process, the project team together with the hospital board, set out a number of design principles or criteria to be taken into account in the system’s design:

- First, the IFMS protocol would have to be developed in consultation with the various stakeholders and should align with the ambitions of the AMC regarding the integration of the IFMS procedure with the existing procedure of annual reviews.
- Second, the MSF tool should cover the broad range of competencies as described in the competency framework used by the Dutch Central College of Medical Specialists (CCMS) [27]. Also, the tool should be scientifically sound, easy to use and avoid administrative burden for both the physician being evaluated as well as his/her assessors. Other feedback tools known to the hospital’s physicians were to be included as well.
- Third, the coaching approach to discuss the feedback report had to optimally facilitate physicians’ professional development. In practice, this meant: facilitating interpretation of feedback data, supporting physicians in setting developmental goals in the context of their own practice, and develop a route to improvement and change.
- Fourth, the coaches needed to be skilled, trustworthy and have close affinity with the medical workplace. Also, as a means to increase physicians’ involvement in the IFMS system, the system needed to be based on the principle ‘for physicians, by physicians’. For these reasons, coaches should be recruited among the physician workforce (peer coaches).
- Fifth, the redesign process would be subjected to evaluation and research. It was thus decided to pilot-test the IFMS procedure in one department, the neurology department. Evaluation results would serve as input for redesign or adjustment of the procedure. After piloting the procedure and recruiting and training peer coaches, the procedure would be evaluated in two other departments at the start of hospital-wide implementation. Thereafter, the implementation process would be scaled up to include all physicians. Last, the handling of the IFMS procedure would ultimately have to be transferred from the (temporary) project group to the organization.

## Results

In this section, we will subsequently describe the five steps of developing the IFMS system in the Academic Medical Center (i.e. IFMS protocol, MSF tool, coaching approach, coaches, and hospital-wide implementation).

### IFMS protocol

In consultation with the various stakeholders and based on incremental evaluation (see further), the system was written down in detail in the IFMS protocol. Since the AMC was the first Dutch academic medical center to present its solution to the new IFMS requirement, the AMC offered its protocol for review to the (national) medical registration authority (RGS). The RGS sanctioned the protocol. Next, the protocol was approved by the hospital’s highest medical leadership and the Works Council, and finally ratified by the hospital’s Executive Board. Upon request, the research team generously shared information about the IFMS protocol with individual hospitals and through various professional networks, i.e. by presenting the protocol in multiple meetings of the Dutch Federation of Medical Specialists, in particular an invitational meeting for University Medical Centers medical staff representatives in November 2018.

**Fig. 1:**
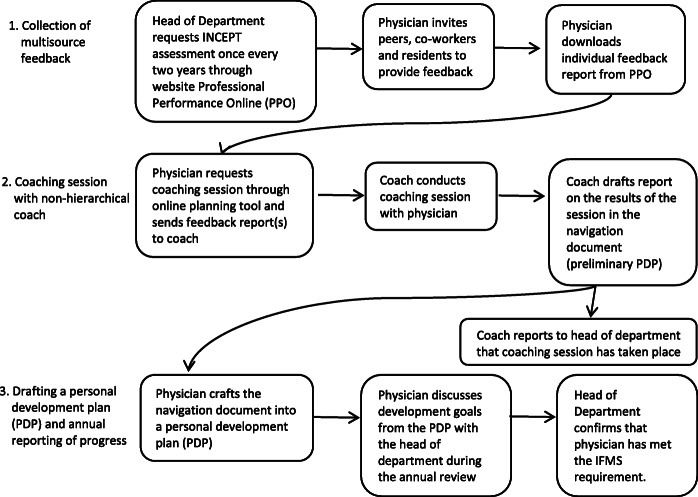
Timeline IFMS trajectory in the Amsterdam Academic Medical Center (AMC)

The protocol describes the process of collecting MSF data, the (planning of the) coaching session, the documentation of the session, drafting the personal development plan (PDP), and the reporting and discussion of the PDP with the (hierarchical) peer superior (see Fig. 1). It contains specific articles reflecting ‘the middle ground’ between what is mandated by law and what is preferred in terms of prioritizing and promoting professional growth. Notably, the protocol contains the ‘negotiated compromises’ related to confidentiality of the coaching session and (non-)disclosure of its findings. Specifically, the protocol determines the face-to-face (peer) coaching session as strictly confidential and the physician is invited to bring any (performance) topic to the table for discussion. Also, the protocol prescribes that the resulting navigation document – a preliminary PDP -(see Fig. 1) remains confidential; it is up to the physician whether or not to share this document with others, including his or her superior. Following the protocol, the only thing the coach is allowed to report to the physician’s superior is that the coaching session has taken place. Physicians may craft their navigation document into a final PDP. In contrast to the navigation document, the final PDP which is modified and approved by the physician, is sent to the superior for discussion during the annual review. The superior will confirm and note in the physician’s HR file that the specialist in question has met the IFMS requirement; this documentation serves as proof of compliance to be forwarded to the authorities, the RGS.

Some articles in the protocol show how the AMC chose for exceeding the minimum legal requirements in favor of facilitating professional development.

- First, instead of once every five years as required by law, it was decided to facilitate participation in the IFMS procedure twice every five years.
- Second, for intervening years, the protocol recommends to additionally discuss the progress of the PDP with peers during a peer group meeting, under supervision of an external coach.
- Third, in addition to the required MSF data (see further) all specialists are expected to also submit other available systematically collected performance information. In particular, physicians should submit their feedback reports on their supervisory and teaching skills as collected through the well-validated System for Evaluation of Teaching Qualities (SETQ) [28].

### MSF tool: INCEPT

The INCEPT (INviting Co-workers to Evaluate Physicians Tool) questionnaire was selected to collect multisource feedback [23]. The INCEPT had been previously developed by the research group PP&CC and had been investigated for its psychometric qualities and feasibility. The INCEPT covers three domains of professional performance: professional attitude, patient-centeredness and organization and (self-)management. It is a 21 item questionnaire and facilitates respondents’ narrative comments. Collection of feedback takes place in a web-based environment, through which physicians invite 8 peers, 8 co-workers and 8 residents to evaluate their performance and also fill in a self-evaluation. Once the feedback is gathered, on average after four weeks, an individualized MSF report is generated. Physicians can download this report through their personal online profile and send the report to the coach. They are encouraged to also include other sources of feedback such as residents’ evaluations regarding training activities. Optionally, physicians can fill in a reflection form in the same web-based environment in preparation for the coaching session.

### Coaching approach: DANA

#### Development of the DANA

A coaching approach was developed by a psychologist (AN), who had approximately 20 years of experience in coaching physicians. The newly developed approach was based on Appreciative Inquiry practices and solution-focused coaching and was named the Developmental Appreciative Navigational Approach (DANA). The aim of DANA was to optimize the use of each individual physician’s talent, to identify visible and hidden personal qualities and to formulate an overarching professional development goal. To enable the physician to expand on intrinsic motivation and individual qualities and to prevent the coaching session from becoming a tick-box-exercise in which the coach would skim through the feedback, the duration of the coaching session was set at two hours. The session was preferably held in the privacy of the physician’s office, where confidentiality was assured at the start of the session. The session resulted in the formulation of a (short) navigation document summarizing the physician’s qualities and ambitions, a description of one or more identified developmental goals and multiple concrete actions, preferable set in time and place to achieve those goals. An overview of the substantive elements of the DANA is displayed in Table 1.

**Table 1:** Steps of the Developmental Appreciative Navigational Approach (DANA)

Step	Content
1. Introduction	Build a foundation for a trusting relationship.
2. Confidentiality	Discuss the purpose of the coaching session and explain the procedure.
3. ‘In your element’	Explore the ideal work situation, when the physician is able to work in a way that gives energy and brings out the best in him/her.
4. Desired future development	Explore ambitions and wishes for the future and satisfaction with the current range of duties.
5. Performance feedback data	Recognize a pattern in the personal feedback data.
6. Personal qualities	Name qualities and connect with feedback data.
7. Improvement goal	Identify and develop a plan for improvement; use scale questions to gain insight into motivation and self-efficacy.
8. Document	Jointly reflect on the session and provide a written summary of the content of the session and the development plan discussed.

#### Evaluation of the DANA - methods

The newly developed DANA was pilot-tested in the department of neurology; all neurologists participated in an individual coaching session with the experienced psychologist. Within two weeks after the session, a researcher (KL) conducted semi-structured interviews to evaluate how they had experienced the DANA session in the light of their professional development. The interview contained questions about how the session had contributed to their insight into personal qualities and developmental opportunities. In addition, several grading scales were used, such as “I have experienced this session as ‘empowering’ vs. ‘discouraging’/‘appreciative’ vs. ‘judgmental’/‘safe’ vs. ‘unsafe’. Furthermore, specialists were asked how they had experienced the role of the coach and whether they had any objection to discuss the documentation of the coaching session with their superior during their annual review. Lastly, they were asked about the appropriateness of the five-yearly frequency of this coaching session. Interviews lasted for 30–50 minutes and were audio-taped. The content of the interviews was summarized and used as input for adjustment of the procedure. The interview protocol is available as supplementary material.

#### Evaluation of the DANA - results

The pilot test in the neurology department took place from January till March 2017. All 17 neurologists rated the session as highly motivating, appreciative, development-oriented, concrete and valuable. They characterized the session as open and safe. For most of them, the session did not reveal new insights about their performance. Rather, it confirmed existing ideas, or was instrumental in rendering, accepting or internalizing the feedback received. The session did however deepen their insights in their developmental opportunities and improvement goals. For all neurologists, at least one improvement goal was formulated. They reported to be highly committed to and motivated for this goal. The role of the coach was greatly appreciated and unanimously rated with a minimum score of 8 out of 10. Some expressed to be interested in additional coaching sessions to follow up on the defined development goals. Most neurologists reported to consider peer coaches to potentially also be good coaches, although some expressed their preference for a professional coach. This preference was related to both the confidentiality of the coaching session and the expertise of a professional coach. Criticism was expressed in terms of the disclosure of the coaching document to their superior, i.e. the Head of Department, as this could hamper the openness during the coaching session. The neurologists supported the proposal to schedule a coach session every two years, instead of once every five years as required by law. Based on the evaluation of the pilot test the protocol was adjusted.

### Coaches

#### Recruitment and training of coaches

Given the importance for coaches to be trustworthy, and bearing in mind the requirement of budget neutrality, coaches were recruited among the hospital’s own physician workforce. The Executive Board sent out a letter to all senior medical leadership – i.e. chairs of the organizational divisions- in which they were requested to nominate a number of potential coaches in proportion to the number of physician staff working in the division. Qualities such as good communication and empathic skills were emphasized as crucial selection criteria. The coaching job would take approximately 30–40 hours per year for 8-10 coaching sessions, including preparation and drafting the navigation report. At least once a year, coaches would participate in a two-hour meeting with other coaches to exchange experiences and improve skills. The appointment as a coach was set for the duration of three years. Participation in a training guided by the experienced psychologist was mandatory. The training took 8 hours and consisted of information transfer, direct teaching, experiential personal development exercises and coaching skills practice, using role play. For participation in the training, 8 accreditation points were awarded.

#### Evaluation of coaches

Peer coaches were recruited among the physician workforce in July 2017. In October 2017, 10 peer coaches were trained by the psychologist (AN). Subsequently, the project team planned coaching sessions for them within two departments, neonatology and rehabilitation medicine. Within two weeks after the coaching sessions in these departments, two researchers (BB, RS) conducted semi-structured interviews with physicians to evaluate the session and the role of the coach, using a shortened version of the pilot interview protocol. Interviews lasted for 15–20 min and were audio-taped. The content of the interviews was again summarized and used as input for adjustment of the procedure.

In total, 15 physicians were interviewed; their experiences were comparable to those of the neurologists in the pilot phase. However, a number of physicians noted that the emphasis in the coaching session had been very much on the appreciation of positive qualities, leaving less room and attention for the formulation of developmental goals and drafting a plan for attaining these goals. In response to this, more attention was paid to strengthening the developmental orientation of the coaching conversation in the DANA training and the meetings with the coaches, by paying special consideration to the factors which may both impede and enable progress and success, setting timelines and considering how to measure success. Once again, the evaluation results showed that physicians sometimes had a preference for a professional non-peer coach. Based on these results, the inclusion criteria for the recruitment of additional coaches were extended to also include psychologists and professionals who otherwise had insight into the physician workplace. A list of external coaches was compiled to be able to refer physicians to a professional coach for additional sessions if desirable or necessary, which could be funded from their personal budget.

### Hospital-wide implementation

#### Gradual scale-up

After (initial) implementation in the departments of neurology (pilot) and neonatology and rehabilitation medicine in 2017, the IFMS procedure was implemented in another 6 departments in 2018. The project team contacted the heads of these departments and informed the medical staff about the IFMS procedure. Practical matters such as the management of MSF through the web-based environment were entrusted to secretary staff, who were provided with manuals and templates. Upon request, heads of department were provided with a training on how to follow-up on the coaching session during the annual review in a positive and development-oriented manner. In October 2018, the group of coaches was expanded with 9 coaches. In the same period, an online planning tool for efficient scheduling of sessions was introduced. Coaches registered their availability and provided a profile text about themselves, so that physicians could make an informed decision for a coach. To support coaches in their development as a coach, coach meetings were organized which were supervised by the psychologist. By the end of 2018, 96 specialists had taken part in the IFMS procedure.

#### Transfer to HR department

In May 2019, the design, evaluation and implementation of the IFMS system was completed and the procedure could be embedded into the organization. The operational responsibility of the IFMS procedure was transferred from the research group PP&CC to the hospital’s Human Resources department. The research group continued to support and advise the HR staff on content matters, and also continues investigations into the follow-up on coaching sessions. The HR department supported continuous recruitment and training of coaches, as well as the scheduling of coach meetings and the monitoring of the degree of participation and lead time on department level. In 2019, another 12 departments took part in the new system and the group of coaches was brought to final strength with the addition of 11 coaches, resulting in a total number of 30 coaches. By the end of 2019, 162 out of 624 physicians in the hospital had completed the IFMS procedure.

## Discussion

In this paper we reported on a design-based research (DBR) development of a new performance appraisal system that serves both physicians’ professional development needs and mandatory accountability, or re-registration requirements. The new system reflects the compromises made in dealing with the perceived conflicting nature of development and accountability purposes. Characteristics that appeared crucial and non-negotiable for the facilitation of professional development were an appreciative and development-oriented performance appraisal process, the trustworthiness and skills of the peer coaches and a non-disclosed appraisal output. The DBR approach contributed to the successful implementation and positive reception by physicians, organizational leadership and regulators. We will discuss our findings in more detail below by elaborating on (1) the combination of MSF and coaching in performance appraisal, and (2) professional regulation and performance appraisal in the organizational sphere.

### The combination of MSF and coaching in performance appraisal

In many western healthcare systems (e.g. the USA, Canada, the UK, the Netherlands), initiatives have been developed to facilitate reflection on MSF results [12, 29, 30]. In this paper we reported on the development of a new coaching approach (DANA) to guide peer-to-peer conversations following MSF. To our knowledge, this approach is among the first positive psychology coaching interventions that have been specifically developed for practicing physicians participating in an MSF program in the context of revalidation. The evaluation results of the pilot and the initial implementation showed that physicians highly appreciated the session and the coaches. Peer coaches were considered trustworthy given their familiarity with the working context. Nevertheless, some physicians expressed a preference for an external professional coach given their specific skills, ample coaching experience and relative independence of “the system”. The latter seemed particularly true for the more senior physicians in leadership positions. Both expertise and confidentiality were considered of special importance because of the potential sensitivity of the topics that were discussed during the coaching session. Although we did not systematically collect these data, anecdotal evidence showed that physicians struggled with high workloads, organizational changes, well-being problems or issues related to interpersonal dynamics with colleagues and managers. This is in line with research on the relationship among physicians’ workload, social support and well-being [31–33], highlighting the need for research that strengthens the evidence-based underpinnings of the positive psychology approach in organizations [34].

### Professional regulation and performance appraisal in the organizational sphere

The results as reported in this paper should be considered within the dynamics of the organizational sphere of the hospital setting. With the advent of revalidation, organizations have become intermediaries in the relationship between physicians and regulatory authorities, enacting regulatory processes on their behalf and extending regulatory surveillance and oversight at local level [35]. Within this organizational sphere, however, complex relational and governance issues already exist. In this paper, the challenge of effectively combining the formative coaching session with the existing hierarchical annual review with the peer superior deserves special attention. The initial proposal of offering physicians a personal coaching session which outcomes were then to be presented to their superior for the purpose of supporting implementation of the PDP in practice, was not deemed feasible. Understandably, knowing that the results of a personal coaching session will be shared with superiors, likely inhibits the agenda setting, openness and depth of a conversation. In practice, this could lead to suboptimal sessions when areas in need of improvement or personal ambitions that conflict with the strategic objectives of the organization, would remain underexposed. This potential adverse effect should always be considered in light of the formal requirements which do not mandate disclosing results to any authority. In our case, it was decided that safety and confidentiality should take precedence over involvement of organizational leadership. This may indicate physicians’ lack of trust in the organization. Although in the literature it has been pointed out that the traditional professional-manager dichotomy is no longer a valid reflection of contemporary professions and organizations [36–38], the experience of such a dichotomy may still be present, especially within hierarchical academic medical centers. The current system’s design, where physicians are encouraged to share their performance results and coaching session’s report but may choose not to, can be considered a compromise. The future will have to show how this turns out in practice. We believe the negotiated compromise is an important contribution to meaningful conversations.

### Strengths and limitations

In accordance with the principles of DBR, the design and implementation of a new system for performance appraisal was carried out as a cyclic process of design, evaluation and redesign, in an authentic real-life setting, in which the researchers, designers and practitioners interacted frequently to share their ideas. The DBR approach allowed us to propose, test and amend solutions for the challenging goal of satisfactorily serving the different purposes of accountability and professional development. The resulting system, as reported in this paper, reflects the various compromises that were reached through extensive deliberations. For the success of the system, during the design process we constantly kept in mind the importance of the quality of the feedback conversation. We did this by developing a new coaching approach and ensuring a thorough evaluation, first when the conversation was conducted by a psychologist and later again when the conversation was conducted by a peer coach. The main limitation of this DBR project relates to tracking the actual impact of the performance appraisal process on physicians’ subsequent professional performance and development. Given the size and lead time of the project and our focus on a solid design solution, an extensive and complete impact analysis is not yet available. Efforts to secure reflexive monitoring are still underway. Another consideration is the fact that this project took place in the local setting of one Dutch academic medical center, so the proposed design solution may not fit other contexts. However, several other (academic) medical centers have shared with the authors that they copied our protocol or used it as a basis to build their own. This suggests that the design is at least to some extent transferable to other settings. Nevertheless, we did undertake steps to stimulate a broader relevance than the local situation by including a detailed description of the context of the project and formulating clear design principles. Similar to many other DBR projects, the roles of researcher and designer were fulfilled by the same persons. In order to prevent the findings of the study to be influenced by the researchers’ biases, two researchers from another institution (AS and SH) were involved in the research process.

### Recommendations for practice

There are a number of practical matters that should be taken into account when designing and implementing a performance appraisal system in the hospital organization. Efforts at different times in the design and implementation process and at different organizational levels are necessary. First, it is crucial that the system will not add too much administrative burden to physicians and the organization in general, for example by making use of existing and for physicians familiar infrastructures to collect feedback data or plan coaching sessions. Second, the timing of the appraisal process within a department needs to fit in with other ongoing projects, so that physicians are supported in their commitment to provide meaningful feedback to their fellow physicians. Third, it is important to ensure that peer coaches build coaching expertise by conducting sufficient sessions per year. The challenge for organizations is to find workable arrangements to combine physicians’ time commitments to coaching and their clinical responsibilities. A combination of peer coaches, non-peer coaches and external coaches may be one way to deal with this. Fourth, after the system’s initial implementation, the organization needs to secure reflexive monitoring. This may be considered a typical responsibility of the HR department, showing the organization’s commitment to a high performing medical staff.

### Future research

To ensure that the performance appraisal system will indeed contribute to physicians’ professional performance and development, it is crucial that the system becomes routinely embedded in physicians’ organizational and professional contexts. A theory that may serve as a sensitizing tool for longitudinal monitoring efforts is the normalization process theory (NPT) [39, 40]. The framework originating from this theory facilitates systematic exploration of why some processes lead to a practice becoming successfully (or not) embedded (i.e. normalized) and sustained, by attempting to understand the intervention in relation to the everyday practice of those involved. There are four main components to NPT which may be the foci of monitoring efforts: coherence (i.e. physicians’ sense-making of the performance appraisal process in the context of revalidation); cognitive participation (i.e. commitment and engagement of physicians); collective action (i.e. the work physicians have done to make the performance appraisal process function); and reflexive monitoring (i.e. the evaluative work researchers and stakeholders do to assess and understand the benefits and costs of the performance appraisal process). For the last component, it is important to bear in mind the nature of the topics discussed during coaching sessions. Monitoring efforts could include person-centered measures related to work engagement, well-being and interpersonal communication, complemented with more distal outcomes of coaching related to the wider organization, such as team climate and overall hospital culture. In this regard, the proposed group meetings to discuss the progress of the PDP with peers, led by an external coach, may offer additional opportunity for gaining insight into how the new system can contribute to performance change. In the event that the appraisal procedure and the annual review procedure remain separated, hospitals need to be – in some way – informed about the content or results of the coaching sessions. This is imperative, as hospitals and physicians bear a joint responsibility for the quality of care.

## Conclusions

In this study, we designed a new performance appraisal procedure for physicians undergoing re-registration that would successfully comply with both accountability requirements as well as fulfill the need for professional development. The study took place in the local context of a large academic medical center in the Netherlands and the findings maybe translated into design guidelines broadly applicable in other hospital settings, and even healthcare systems. In the context of revalidation, professional performance and development can be supported when the appraisal process is appreciative and development-oriented, when the coach is skilled and trustworthy and when the physician has control over the disclosure of the output of the process to others.

## Supplementary Information

**Additional file 1.** Interview protocol for the evaluation of the DANA / coaching session

## Data Availability

The dataset analyzed during the current study is available from the corresponding author on reasonable request.

## Abbreviations

CPD: Continuing Professional Development
MoC: Maintenance of Certification
MSF: Multisource Feedback
DBR: Design-Based Research
IFMS: Individual Functioning Medical Specialists
INCEPT: INviting Co-workers to Evaluate Physicians Tool
SETQ: System for Evaluation of Teaching Qualities
DANA: Developmental Appreciative Navigational Approach
