# Supplementary material for: Designing a system for performance appraisal: balancing physicians’ accountability and professional development
Source: BMC Health Serv Res. 2021 Aug 12;21:800. doi: 10.1186/s12913-021-06818-1 (PMC8359079; doi:10.1186/s12913-021-06818-1)
Supplement: Supplementary file 1 — Additional file 1. Interview protocol for the evaluation of the DANA / coaching session [file 12913_2021_6818_MOESM1_ESM.docx]

Appendix. *Interview protocol for the evaluation of the DANA / coaching session*

| The purpose of this interview is to evaluate your recent coaching session. Based on the results, an advice will be formulated regarding the implementation of coaching sessions for all medical specialists. Central to the evaluation is your experience with the Developmental Appreciative Navigational Approach (DANA) used in the coaching session.   1. Looking back on the session, which three words best describe your experience?   ……………………………………………………………………………………………………………   1. a. Did the coaching session provide you with a (better) insight into your qualities as a   medical specialist?  surely / slightly / hardly / not at all  b. Can you indicate what contributed to (not) gaining this insight?    ……………………………………………………………………………………………………………   1. a. Did the coaching session provide you with a (better) insight into your areas for   improvement as a medical specialist?  surely / slightly / hardly / not at all  b. Can you indicate what contributed to (not) gaining this insight?  ……………………………………………………………………………………………………………   1. Can you characterize the coaching session as you experienced it on the following scales (put an X on the line):   Discouraging Empowering  l_________________l_________________l__________________l________________l  Normative Educational  l_________________l_________________l__________________l________________l  Energy-consuming Energizing  l_________________l_________________l__________________l________________l  Unsafe Safe  l_________________l_________________l__________________l________________l  Judging Appreciative  l_________________l_________________l__________________l________________l  Focus on weaknesses Focus on strengths  l_________________l_________________l__________________l________________l  Closed Open  l_________________l_________________l__________________l________________l  De-motivating Motivating  l_________________l_________________l__________________l________________l  Worthless Valuable  l_________________l_________________l__________________l________________l  Superficial Profound  l_________________l_________________l__________________l________________l  Tense Relaxed  l_________________l_________________l__________________l________________l  Little development-oriented Highly development-oriented  l_________________l_________________l__________________l________________l  Abstract Concrete  l_________________l_________________l__________________l________________l  Theoretical Practical  l_________________l_________________l__________________l________________l   1. a. Have you been able to draft a preliminary personal development plan (PDP) during the   coaching session?  Yes / No  b. Does the preliminary PDP contain the most important issues related to your performance  and development?  Yes / No  c. On a scale from 1 to 10, to what extent do you feel committed to realizing / implementing  this PDP?  1 = I don’t feel committed at all  10 = I feel highly committed My grade: …………    d. Expressed in percentages, how do you estimate the chance that you will have realized  your PDP in one year?    ………….%  e. Are there any objections for you to discuss this PDP with your superior during the annual  review?  Yes / No  If yes, can you explain your objections?  ……………………………………………………………………………………………………………   1. The coaching session is in principle a one-time session, with a follow-up during the annual review (discussion of the PDP). How do you feel about this as a starting point?   ……………………………………………………………………………………………………………   1. Do you consider the IFMS procedure (multisource feedback + coaching session + PDP) relevant for re-registration?   Yes / No  Any explanation?  …………………………………………………………………………………………………………   1. If you could summarize the coaching session in an image or a metaphor, which image or metaphor would you choose?   ……………………………………………………………………………………………………………   1. Based on your experience with the coaching session, what would be your advice on coaching sessions in the Amsterdam Academic Medical Center (AMC)?   ……………………………………………………………………………………………………………  Thank you for your cooperation. This data will be processed anonymously. Results will be reported in such a way that they cannot be traced to individuals. |
| --- |
